# A database of refractive indices and dielectric constants auto-generated using ChemDataExtractor

**DOI:** 10.1038/s41597-022-01295-5

**Published:** 2022-05-03

**Authors:** Jiuyang Zhao, Jacqueline M. Cole

**Affiliations:** 1 Cavendish Laboratory, University of Cambridge, J. J. Thomson Avenue, Cambridge, CB3 0HE UK; 2 ISIS Neutron and Muon Source, Rutherford Appleton Laboratory, Harwell Science and Innovation Campus, Didcot, Oxfordshire OX11 0QX UK; 3 Department of Chemical Engineering and Biotechnology, University of Cambridge, West Cambridge Site, Philippa Fawcett Drive, Cambridge, CB3 0AS UK

**Keywords:** Optical physics, Optical physics, Metamaterials, Nonlinear optics

## Abstract

The ability to auto-generate databases of optical properties holds great potential for advancing optical research, especially with regards to the data-driven discovery of optical materials. An optical property database of refractive indices and dielectric constants is presented, which comprises a total of 49,076 refractive index and 60,804 dielectric constant data records on 11,054 unique chemicals. The database was auto-generated using the state-of-the-art natural language processing software, ChemDataExtractor, using a corpus of 388,461 scientific papers. The data repository offers a representative overview of the information on linear optical properties that resides in scientific papers from the past 30 years. Public availability of these data will enable a quick search for the optical property of certain materials. The large size of this repository will accelerate data-driven research on the design and prediction of optical materials and their properties. To the best of our knowledge, this is the first auto-generated database of optical properties from a large number of scientific papers. We provide a web interface to aid the use of our database.

## Abstract (summary)

**Table:** 

Measurement(s)	refractive indices • dielectric constants
Technology Type(s)	natural language processing

## Background & Summary

Optical materials are essential components of modern optical devices. Scientists have increasingly found applications of optical materials in information technology, lasers and optical telecommunications. Various kinds of materials have been used for making optical elements, based on how they interact with electromagnetic waves. Glassy materials with good light transmission are often used as optical lenses or windscreens. Materials with certain light absorption characteristics can be used to make optical filters. As a quantitative measure of how a material interacts with light, the optical properties of materials are of great importance. Access to a knowledge base of optical materials and their properties would be particularly valuable for optical and optoelectronic applications, such as organic light-emitting diodes, image sensors, and projector lenses^1^. Given the increasing demand for advanced optical device applications of this nature, extensive efforts are being made in this field to develop novel materials; those that display extreme optical properties or provide precise estimations of certain types of optical properties are of particular interest^2,3^.

Traditional experimental and computational methods of developing new materials tend to rely on scientific intuition and are very consuming in time and resources^4,5^. Indeed, the average “molecule-to-market” timeline for a new material is around 20 years, which is far too long for the industry^6^. The Materials Genome Initiative (MGI) was created in 2011 out of concern for this issue and related matters. The MGI sought to stimulate ways to accelerate the design and discovery of new materials. The concurrent emergence of data science at that time fostered a massive opportunity for researchers to develop pipelines for data-driven materials discovery. The idea of “materials-by-design” using machine-learning methods has now achieved huge success in multiple subjects, from designing new drugs^7^ to developing functional ternary oxides^8^. However, the data-driven discovery of new materials is not feasible without the premise of a comprehensive database.

Since the initiation of the MGI, many projects that span different fields have been initiated to build extensive databases for use in materials discovery. Yet, current databases are mostly based on theoretical calculations, one of the most prominent being the Materials Project^9^. A few databases are starting to form from the output of high-throughput experimental pipelines, such as the open-database on inorganic thin-film materials generated by the National Renewable Energy Laboratory, NREL^10^. However, these two types of data have certain shortcomings. The theory used in computational calculations has reduced applicability outside the materials space for which the theory has been parameterised. Experimental databases usually encounter a limited sample diversity problem and the restricted nature of the metrological methods used tends to constrain the type of data that are sourced.

Another way to realise materials-property databases is to auto-generate them by mining data from the scientific literature. Over the past few decades, an ever-increasing number of academic papers on optical materials have been published in electronic form. This makes the extraction of material structures and optical property data from unstructured text feasible. This study demonstrates that a large {material - property} database of refractive indices and dielectric constants can be auto-generated for optical research, from the scientific literature. We show that this database achieves satisfactory precision and diversity for these two optical properties. To the best of our knowledge, this is the first material database of optical properties that has been auto-generated from the literature. The state-of-the-art natural language processing software, ChemDataExtractor^11^, was used for this work, with minor modifications, to meet the needs of the specific domain of optical properties. The auto-generation process of our database comprised four steps: article retrieval, relationship extraction, data post-processing, and technical evaluation. A certain noise level was apparent in the initially formed database, but proper filters were constructed to filter out the noisy outliers. The resulting database exhibits a large diversity of optical materials. As such, it has wide application prospects in the data-driven materials discovery of optical materials and the precise estimation of optical properties.

## Methods

### Article retrieval

The basic web scrapers of ChemDataExtractor^11^ were modified and adapted for this work in order to scrape the full-text of scientific papers from the Royal Society of Chemistry (RSC), Elsevier, and Springer Link, within the modes of publisher authorisation. The scrapers use Python HTTP client libraries “urllib” and “requests” to send requests to a server at the publisher and collect resent information. The selenium package of Python was used to simulate human interactions with the RSC website since the RSC website does not provide direct access through HTTP requests. Selenium browsed each page of the article search result and searched for full-text hyperlinks to download full-text HTML files. An Application Programming Interface (API) was provided by Elsevier for users to perform complete data mining. A HTTP PUT request was sent to Elsevier to obtain the Digital Object Identifier (DOI) information of papers, and papers were downloaded through links provided by Elsevier ScientificDirect APIs. The scraping from Springer Link was performed in a similar manner to that of Elsevier, but only open-access papers were allowed to be scraped.

In addition, these requests involved query search keywords, “refractive index” and “dielectric constant”. The range of publication years for papers was set to be 1990–2020. To this end, a total number of 186,196 papers were downloaded for query “refractive index” and a total number of 230,980 papers were downloaded for query “dielectric constant”.

### Document processing

Once ChemDataExtractor^11^ has obtained scientific papers in HTML/XML format, the toolkit takes advantage of its “reader” packages to standardise the document into plain text. Since the original files have hierarchical structures, where different tags indicate different sections of the article, the reader makes use of these tags to segment the document into different domains. For example, when the reader encounters a <title> tag in a HTML file, it will recognise the content within this tag as the title of the article and extract the raw text as a Python bytes object. After completing this segmentation process, fragmented data of different text sections are merged together to provide a simplified, cleaned document consisting of title, abstract, headings, paragraphs, figures, table elements, and so on. Since the journal citation information is essential for the database construction process, a “metadata” function is also embedded in the reader to retrieve the DOI, journal, publication date and title information of the paper.

### Natural language processing (NLP)

Natural language processing (NLP) plays a key role in modern information extraction processes, as it programs the computer to teach it how to analyse textual data based on human knowledge. A comprehensive NLP toolkit is embedded in ChemDataExtractor^11^ for its specialist application to the physics, chemistry, and materials-science domains. It includes four noteworthy stages: tokenisation, word clustering, part-of-speech (POS) tagging, and chemical named entity recognition (CNER). Tokenisation splits the sentence into a sequence of its constituent parts (called “tokens”), such as individual words, digits and punctuation. Word clustering groups words into clusters to reduce the computing-power cost of POS tagging and CNER. POS tagging assigns to each token a POS tag, such as “noun”, “verb” or “adjective”, that describes its syntactic property. CNER aims to identify all valid chemical named entities across the document. The performance of CNER directly determines the precision of the resulting database.

### Relationship extraction (RE)

**Fig. 1:**
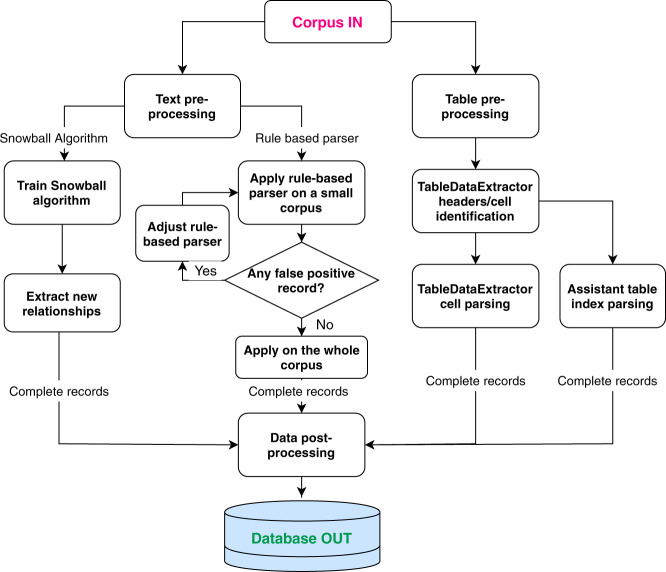
Pipeline of relationship extraction of ChemDataExtractor.

After the document processing and core NLP stages, extracting relationships between materials and properties is the most essential step of auto-generating the target database. ChemDataExtractor version 1.5 was used to mine relationships from text, using its POS taggers and CNER system^11^, as well as a semi-supervised Snowball algorithm^12^ that has been modified to enable quaternary relationship extraction of chemical, property, value, unit tuples^13^. Minor modifications to ChemDataExtractor were made to extract relationships specifically for the subject database, including a rule-based parser template for optical properties. This text extraction was complemented by the use of ChemDataExtractor version 2.0^14^ which was used to mine relationships from tables. Thereby, a new table processor, TableDataExtractor, is a Python library that has been built into ChemDataExtractor 2.0^14^. It standardises the format of tables in order to parse its contents. An assistant table parser with a looser logic was written for this study, in order to facilitate this relationship extraction from tables. Figure 1 shows an overview of the entire relationship extraction pipeline of the ChemDataExtractor used in this study. In the following subsections, the different parsing routes of this pipeline will be described in more detail.

### Text mining

A key idea within the text-mining operation of ChemDataExtractor^11^ is the use of rule-based parsers to enable string matching, which is facilitated by the use of regular expressions in Python. A rule-based parser consists of two parts: the parsing elements and parsing grammar rules. The parsing elements are a set of Python functions that wrap the regular expressions to help write grammar rules. Some examples of the parsing elements are shown in Table 1.

**Table 1:** Examples of *parsing elements* from ChemDataExtractor.

Elements	Function that matches
I	a token (case-insensitive)
W	a token (case-sensitive)
T	a certain POS tag
R	with regular expression
And	all in a given order
Or	either or
Any	any character
ZeroOrMore	a token ≥ 1 times
SkipTo	any character until a token (inclusive)

For example, the exact word “cake” will be matched but not “Cake” when encountering the expression “W(‘cake’)”. The expression “SkipTo(W(‘cake’))” will skip any characters from the start of a sentence until reaching the exact word “cake”. The parsing elements are designed to help write parsing grammar rules. That way, users do not need to write complex regular expressions by themselves but they can just call these parsing elements when needed.

By making use of these parsing elements, ChemDataExtractor has a pre-defined property parser template, i.e. a set of hand-crafted grammar rules/patterns to recognise relationships in the sentences. Each pattern is intrinsically a phrase of sentence written in terms of regular expressions. All patterns must have three indispensable parts: a chemical name (<CEM>), a property specifier (<specifier>), and a value (<value>). The <CEM> element is supposed to be recognised by the CNER system. The <value> element is associated with a set of pre-defined regular expressions for capturing numbers in sentences. And the <specifier> is a collection of words defined by the user that captures possible property names, such as “refractive index” or “R.I.”. Finally, normal words are filled in between these three parts to form a complete pattern.

**Fig. 2:**
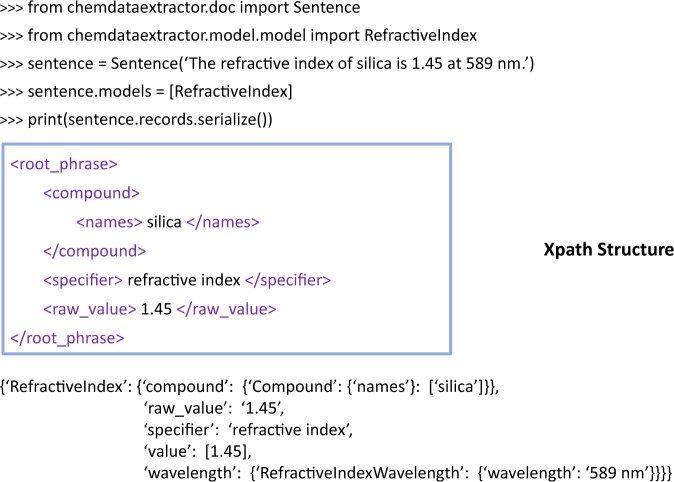
Pseudo-code demonstration of the Xpath parsing process of ChemDataExtractor.

Figure 2 shows an example of a rule-based parser for ChemDataExtractor that was used in this study. “Sentence” is the function, including tokenisation and POS tagging, that pre-processes the sentence. “RefractiveIndex” is a ChemDataExtractor model that aggregates the CNER system, the user-defined property specifiers and the grammar rules. Assigning a “RefractiveIndex” model to the sentence enables ChemDataExtractor to interpret it via an Xpath tree and match the sentence with grammar rules. If the sentence matches a grammar rule, e.g., “I(‘The’) + <specifier> + I(‘of’) + <CEM> + I(‘is’) + <value>”, and all <CEM>, <specifier> and <value> are found, the record will be generated in the form of a Python dictionary. In addition, the property model of ChemDataExtractor offers great flexibility in allowing customised information to be extracted. For instance, it can extract information about a measurement attribute for a key property whose value depends upon that of the attribute. A specific example of such a case is the wavelength information that is associated with a refractive index, which is contained in the sentence “The refractive index of silica is 1.45 at 589 nm”. A “wavelength parser” was created and embedded in the “RefractiveIndex” model to extract this dependent wavelength information within the sentence. Thus, the final record shown in Fig. 2 also contains a “wavelength” attribute that indicates the wavelength information.

The set of grammar rules used in this study was a modified version of a property parser template in ChemDataExtractor. This template has more than 200 pre-written grammar rules for text parsing. The following modifications to this property parser template were made in order to tailor it to the optical properties sought in this study:

- The parser template was adjusted into a dimensionless version since refractive index and dielectric constant are dimensionless quantities. For example, a unit “blacklist” was created and used to prevent ChemDataExtractor from extracting values that were followed by units, i.e., helping to mitigate the extraction of erroneous values.
- Regular expressions were added to capture numbers in the form of scientific notation, e.g., R(“\^\d+(\.\d+)?(x|\*|·)?(10|E|e)[\---^~~^]?\d+\$”)
- New synonym words were added to enrich the grammar rule dictionary, e.g., the synonym of “have” could be “own”, “possess”, “carry”, “retain” etc.
- A “word blacklist” was added. This corrects for the situation where a rule-based parser might otherwise be disturbed by certain words, to afford an incorrect tuple. For example, an incorrect tuple such as {silicon, refractive index, 6} might be extracted from the sentence “The refractive index of silicon can be found in Table 6.”. A “word blacklist” was constructed to exclude any value that is immediately followed by a word therein; words such as “Table” and “Fig.” were added. As such, this blacklist will preclude this incorrect tuple from being generated since the value associated with Table would not be recognised as a value.
- Minor mistakes in the grammars of the parser template were corrected.

Normally, a more complex set of parsing rules is likely to yield a database of higher precision but lower recall. Thus, to preserve a reasonable balance between precision and recall, the modifications mentioned above were all kept at a modest level. A fully detailed version of the grammar rules and the settings of the property specifier can be found via the link in the Code Availability section. In summary, the rule-based parser is able to extract the {material, property, value} relations, and it is also sufficiently flexible that it can be updated and modified. However, the rule-based parser is strict in its requirement to match the correct contents, and it can fail when there is a minor mismatch between rules and the sentence. To allow for cases where such failures happen, the Snowball algorithm^12,13^ within ChemDataExtractor was employed to assist the text-mining process.

The Snowball algorithm uses probabilistic string matching as its core method, in contrast to the deterministic nature of rule-based parsing. Thus, small mismatches between stored grammar rules and the target sentence are allowed. Snowball therefore offers a nice complement to rule-based parsing for string matching. The Snowball algorithm involves three main steps: seed generation, sentence clustering, and new relationship extraction. Unlike the rule-based parser, whose grammar rules were manually written by a human with their experience and knowledge of linguistics, the Snowball algorithm was trained on a small corpus of papers and the selected patterns, the “seeds”, were cached. An active learning routine engaged a human to tell the computer which of the sentences within the document possessed a chemical name, a specifier, and a value in a correct relationship. The selected sentences were classified into clusters based on their textual similarity. For each cluster, a pattern was assigned a confidence score from the sentences within it, based on their frequencies. When encountering an unseen sentence, Snowball calculated the textual similarities between all patterns and the unseen sentence. If any of these similarities exceed a pre-defined threshold T_sim_, and the product of this similarity and the pattern confidence exceeds the minimum relation confidence T_c_, then the relationship existing in that sentence will be extracted.

The Snowball algorithm features a high precision but a relatively low recall due to a finite size of seeds. To increase the recall without loss of precision, the Snowball algorithm in ChemDataExtractor was modified in this study to include the following two functions: a new cluster generation mechanism and a POS-tag matching algorithm. The new cluster generation mechanism allowed the system to generate new clusters through relationship extraction rather than keeping a fixed number of clusters. The default modified Snowball algorithm within ChemDataExtractor will add to a cluster all accepted sentences whose textual similarities ≥ T_sim_ and update the cluster with a certain learning rate. The new cluster mechanism enables sentences with similarities exceeding a threshold, T_n_, to be added to the corresponding cluster, as before; yet, sentences with similarities between T_sim_ and T_n_ will be stored in a new cluster. The pattern confidence score of the new cluster was calculated based on both the textual similarity and the confidence of the pattern being matched, i.e., the new cluster was never more reliable than the old cluster being matched. The nature of this calculation mitigated a loss in precision.

The POS-tag matching algorithm followed the fact that sentences sometimes express the same meaning but use word synonyms, and that synonymous words are likely to share the same POS tag. The POS-tag matching algorithm will only be implemented for a given unseen sentence, if the token match yields no result. The algorithm compares the POS-tag sequence of the unseen sentence with all POS-tag sequences of the stored patterns. This comparison uses the Python Levenshtein distance package to calculate the string similarity between two POS sequences. If the string similarity reaches a pre-defined threshold T_pos_, the relation within this sentence will be accepted. This threshold was chosen in order to keep a reasonable balance between precision and recall.

Detailed parameter settings of the modified Snowball algorithm that were used in this study are listed in Table 2. The meaning of “Prefix”, “Middle” and “Suffix” are tailored to a study. Here, they refer to the word before the first indispensable element, the collection of words between indispensable elements and the word after the last indispensable element. As an example, the prefix, middle and suffix of the sentence shown in Fig. 2 are [“The”], [“of”, “is”, “at”, “589”, “nm”] and [“.”].

**Table 2:** Parameter settings in the modified Snowball algorithm within ChemDataExtractor that were used in this study.

Parameter	Interpretation	Value
T_c_	minimum relation confidence	0.85
T_sim_	minimum cluster similarity score	0.9
Prefix weight	prefix weight in similarity calculation	0.1
Middle weight	middle weight in similarity calculation	0.8
Suffix weight	suffix weight in similarity calculation	0.1
T_n_	similarity threshold in new cluster generation	0.95
T_pos_	minimum relation confidence in POS match	0.85
Learning rate	Step of updating the pattern confidence	0.005

### Table mining

Tables within documents are particularly rich in data given that they feature condensed sets of semi-structured data. A separate pipeline for extracting data from tables is needed in order to take advantage of these manually-formed data structures. A new table processor for ChemDataExtractor has been built for ChemDataExtractor version 2.0^14^. This is called TableDataExtractor and it consists of two parts: a “Table Cleaner” and a “Table Parser”. The table cleaner is responsible for reading tables from original files, identifying table headers/cells and flattening tables into Python arrays. Once flattened, the table parser will join the contents of each cell into a sentence, and a record will be extracted if it contains both a compound name and a property specifier. Apart from the table parser that is built within TableDataExtractor, a lighter table parser was written for this study, to further assist the data extraction from tables. The assistant table parser takes the flattened Python array from TableDataExtractor as an input and searches through row headers for a compound name, with index i, and through column headers for a property specifier, with index j. If the cell (i, j) value is not null, the (compound, property specifier) value pair will be extracted. This provides some specificity to the typical formats of tables reported in the field of optical materials. In general, many tables possess very complicated structures or formats, and the style of the table is extremely variable to a materials domain. For example, a large number of tables have multiple headers, which brings extra difficulty to recognising the identity of the data in each cell. Some tables only state property specifiers or compound names in the table caption. An even more difficult case is a table where a series of chemical mixtures is described as a function of one or more property; there, its series of fractional compositions is typically listed in a table, as labels, to differentiate each mixture within an overarching set of parent chemicals that are being combined to generate a series of properties. The presentation of this list of compositions is frequently separated from that of their chemical names which may or may not even feature in the table at all. The splitting of compositional values of a chemical from their parent name in this way can make it next to impossible to reconstruct the chemical identity of each mixture using automated CNER. A few examples of this problem are given for a binary and ternary chemical mixture for the purposes of illustration: “Table 2” by Tsierkezos *et al*.^15^; “Table 5” by Baskar and co-workers^16^. Despite these difficulties, TableDataExtractor was still able to provide a very good result for relationship extractions from tables. Detailed analysis on the performance of the generated database from tabular data is provided in the Technical Validation section.

### Data post-processing

The data post-processing stage comprised two steps: normalising the chemical names and cleaning the database. The chemical name normalisation process used a subroutine to convert the extracted chemical names into standard formats. For organic chemicals, it used the NLP tool “Open Parser for Systematic IUPAC Nomenclature” (OPSIN) to convert compound names to their simplified molecular-input line-entry system (SMILES) notation^17^. For inorganic chemicals, the subroutine used the National Cancer Institute’s Chemical Identifier Resolver (CIR) through their Python wrapper, CIRpy (https://github.com/mcs07/CIRpy), to convert the inorganic compound names into the Hill Notation^18^. Only the resulting records whose compound names were successfully standardised were preserved in the database.

Rule-based filters were implemented in order to remove data records that are likely to represent an incorrect record. Thereby, the effect of parsing-method failure on database precision was minimised. The filters carried out the following operations:

- Removal of any record whose refractive index has an extreme value. Since refractive index is a dimensionless quantity, numbers (e.g. years and concentration fractions) that appear within the text or tables might be extracted and assigned erroneously to a refractive index. This filter mitigates such false assignments by applying boundary conditions to constrain refractive indices to realistic values. Thereby, any records with refractive index values larger than 10 or less than 1 were removed. This filter was not implemented for the dielectric constant.
- Removal of records whose compound names are incomplete, contain invalid characters or end with abnormal words. For example, names like “Al_2_O_3_ - ” or “Al_2_O_3_ / ” were removed since the second part of the compound was not successfully extracted. Names that contain “$” or “<” were also removed since these characters are rarely used in chemical names, and names containing these characters are much more likely to arise from imperfections of the chemical named entity recognition system.
- Removal of records that have abnormal specifiers. Such abnormalities most typically arise from mistakes in the document processing pipeline or the presence of symbols in text that have non-standard formats. Whitelists were manually created for the two property specifiers that are the focus of this study, and only records whose specifiers reside in these whitelists were preserved.
- Removal of records containing a refractive index which was extracted from an article whose title features the keywords “binary system” or “ternary system”. This removal accounts for the aforementioned table-reading problem that occurs when a table is describing the property of a binary system or a ternary system.

These rule-based filters were designed by manually checking the general tendencies of the incorrect data for common issues and accounting for such edge cases progressively. The data cleaning process will lead to a finite loss of data; however, it can greatly improve the precision of the database.

## Data Records

The database can be downloaded from Figshare^19^; it has been prepared in three formats: SQL, JSON and CSV. A description of each data record in the database is given in Table 3. “Property type” refers to either the refractive index or dielectric constant of the chemical “Compound” in question. “Normalised name” is the normalised compound name mentioned in the data post-processing section. For an organic compound, this is a string of SMILES. For an inorganic compound, it is a list of lists, and each sub-list contains the symbol and the number of one constituent element. “Specifier” is the word in the sentence or table that was used to represent a physical property. “Raw value” and “Extracted value” refer to the value of a chemical property that was extracted and normalised from a string, respectively. The “Extracted Error” of the latter is also recorded. “Wavelength” is a dependent attribute of the refractive index data record, and “Dielectric loss” is a dependent attribute of the dielectric constant data record. These two dependent attributes were only extracted if their values were mentioned together with the relationship in the sentence. The document from which a data record was extracted is described by four data records: its digital object identifier “DOI”, its “Date” of publication, the “journal” name and the “Title” of the article. A warning flag, “S”, is assigned to a data record if the compound associated with this record only appear once in the database. A warning flag, “O”, is assigned to a data record if the value associated with this record is near to the extreme limit (<10th percentile or >90th percentile) of all records for that compound within the database. The ‘Original sentence’ data field stores the original sentence from which the data record was obtained by NLP-based text extraction.

**Table 3:** Description of data records.

Data	Description	Data type
Property type	Optical property type	String
Compound	Chemical compound name	String
Normalised name	Normalised chemical name	List or String
Specifier	Material property specifiers	String
Raw value	Raw values presented in the article	String
Extracted value	Normalised values by ChemDataExtractor	Float
Extracted error^*a*^	Error extracted with the raw value	Float
Wavelength^*b*^	Measurement wavelength information	String
Dielectric loss^*c*^	Dielectric loss information	String
DOI	Source document DOI	String
Date	Source document publication date	String
Journal	Source document journal	String
Title	Source document title	String
Warning	Warning flags ‘S’ or‘O’	String
Original sentence	Original sentence if extracted from text	String

^*a*^“Extracted error” aim for cases such as “1.553 ± 0.05”.

^*b*^For refractive index data records only.

^*c*^For dielectric constant data records only.

## Technical Validation

The evaluation metrics used in this study are precision, recall, and F-score. Precision is the fraction of correct (true) records among the retrieved records. The word “correct” means that the relationship of that record can be identified by a human when reading the corresponding sentence or table. In contrast, an “incorrect” (false) record suggests that a human cannot deduce the relationship of that record from the corresponding sentence or table. Recall is the fraction of the total number of relevant records that were actually retrieved from the entire corpus of papers mined. F-score is the harmonic mean of precision and recall. The metrics can be expressed in terms of the following formulae:

1 $$\begin{array}{c}{\rm{Precision}}=\frac{{\rm{TP}}}{{\rm{TP}}+{\rm{FP}}}\\ {\rm{Recall}}=\frac{{\rm{TP}}}{{\rm{TP}}+{\rm{FN}}}\\ {\rm{F}} \mbox{-} {\rm{score}}=2\cdot \frac{{\rm{Precision}}\cdot {\rm{Recall}}}{{\rm{Precision}}+{\rm{Recall}}}\end{array}$$

where TP (true positive) is the number of extracted, correct records, FP (false positive) is the number of extracted, incorrect records, and FN (false negative) is the number of unextracted, correct records.

To estimate the precision of our database, 500 data records were randomly sampled from the database for each property to construct a small data set for precision evaluation. For a single data record, it was assigned as ‘True’ if the compound name, property specifier and value correctly revealed the relationship that the sentence or the table indicates and vice versa.

To estimate the recall of our database, 100 papers were randomly sampled for each property from the corpus of articles used in the precision evaluation data set. For a single article, all relationships (TP + FN) that exist in the document were manually sought and found. All relationships and records in the database were compared and used to calculate the recall. The details of the precision and recall validation results can be found in the Supplementary Information (S1-S4).

The precision and recall for the text- and table-based data extraction of refractive indices and dielectric constants are shown in Table 4. The 77.22% overall precision and 74.48% overall recall metrics of our database can be compared holistically to those of other databases which were auto-generated using similar NLP-based approaches for other fields of materials science, although they are few at the moment. Court and Cole^13^ used an earlier version of ChemDataExtractor (v1.3)^11^, and a modified Snowball algorithm^12,13^ to create a database of Curie and Néel temperatures for magnetic materials, and they achieved an overall precision of 73%. Compared to our database, this precision is slightly lower, while our data extraction process involved a further developed Snowball algorithm. Huang and Cole^20^ created a database of five properties for battery materials by using ChemDataExtractor version 1.5, and they achieved an overall precision of 80.0% and a recall of 59.1%. Owing to the implementation of TableDataExtractor^14^ in our data-extraction process and the difficulty of parsing complex hierarchical tables, our database shows a slightly lower precision but a significantly higher recall. To the best of our knowledge, this is the first database generated by ChemDataExtractor that uses all three types of parsing logic (rule-based data extraction from text, data extraction using the Snowball algorithm and TableDataExtractor). Moreover, a clear trade-off can be observed between the precision and recall which is consistent with the fact that the total amount of data being extracted should be proportional to the degree of loosening in the parsing logic. A more strict and complex parsing logic will normally provide a more accurate but smaller database. The data records extracted from tables for the refractive index show a relatively lower precision; its primary cause is a misinterpretation of the property specifier, as is discussed in the error analysis section below.

**Table 4:** Precision and recall of the two optical properties for records extracted from text and tables.

Property	Text Precision	Text Recall	Table Precision	Table Recall	Overall Precision	Overall Recall
n	90.11%	71.58%	71.82%	78.42%	75.15%	77.92%
*ε*	78.64%	59.10%	78.95%	72.73%	78.89%	71.70%

The highly complex nature of natural language processing causes a range of error types in data auto-extraction. The errors found during the precision-calculation process of the subject database can be classified into four main types: name errors, coordination errors, parser errors, and table errors; see Table 5. A more detailed error type of each false positive data can be found in the Supplementary Information (S5). Errors in the chemical name account for 46.5% of the data-extraction errors, which occur mostly due to failures in the CNER system. The CNER system is good at recognising a single compound name, but it performs weakly when dealing with more complex chemical names including, but not limited to, doped chemicals, binary or ternary composites, and very long chemical names. For example, “Fe_2-*x*_Mn_*x*_CoSi (x = 0.1, 0.2, 0.3)”, “50/50 PCPM/PMMA”, and “0.2ZnO-0.1Al_2_O_3_-0.09Bi_2_O_3_-0.6B_2_O_3_-0.01Nd_2_O_3_”. Errors in the chemical name of a material can also occur in tables, especially where the constituents of a composite are described in different columns. The table parser may fail to concatenate the individual parts of such a composite. For instance, the first column of a table might state the generic name of a chemical, while the second column describes its manifold dopant and concentration options. In due course, an improvement in CNER will help improve the accuracy of the database. Since the format of tables is highly various across papers, TableDataExtractor may also fail to convert the original table into a standard Python array or fail to identify headers or cells correctly. Overall, though, table-based errors account for only a modest proportion (9.6%) of the total number of errors. The coordination error in Table 5 refers to mistakes in the coordination-resolution process. This is the process in natural language processing that is built to recognise associations between relationships in a multi-entity sentence. For instance, a human should be able to identify two relationships (silicon, refractive index, 3.4) and (SiO_2_, refractive index, 1.45) from the sentence “The refractive indices of silicon and SiO_2_ are 3.4 and 1.45.”. A successful coordination-resolution process would be able to split this sentence into two sub-sentences: “The refractive index of silicon is 3.4.” and “The refractive index of SiO_2_ is 1.45.” Identification failures in coordination resolution are mostly due to one of the chemical entities not being recognised or mismatches between a sentence and pre-defined grammar rules. Nonetheless, this issue accounts for only 2.6% of the errors in this study. The parser error accounts for all other failures of relationship extraction in text (41.3%) which include, but are not limited to, incorrect property specifiers and incorrect values. The extracted property specifier sometimes refers to an unintended property which carries the same label. For instance, the character “n” was defined as the specifier for the refractive index in the data extraction process of this study; however, “n” can sometimes refer to other properties such as the carrier density, a kinetic parameter, or a lattice parameter. In addition, words that were not defined as the specifier can be extracted incorrectly as the specifier if the document source uses a non-standard character encoding. Incorrect values normally refer to the failure of recognising a number written in scientific notation.

**Table 5:** Individual error sources of the false positive data and their percentages.

Error sources	Proportion
Name error	46.5%
Coordination error	2.6%
Parser error	41.3%
Table error	9.6%

**Fig. 3:**
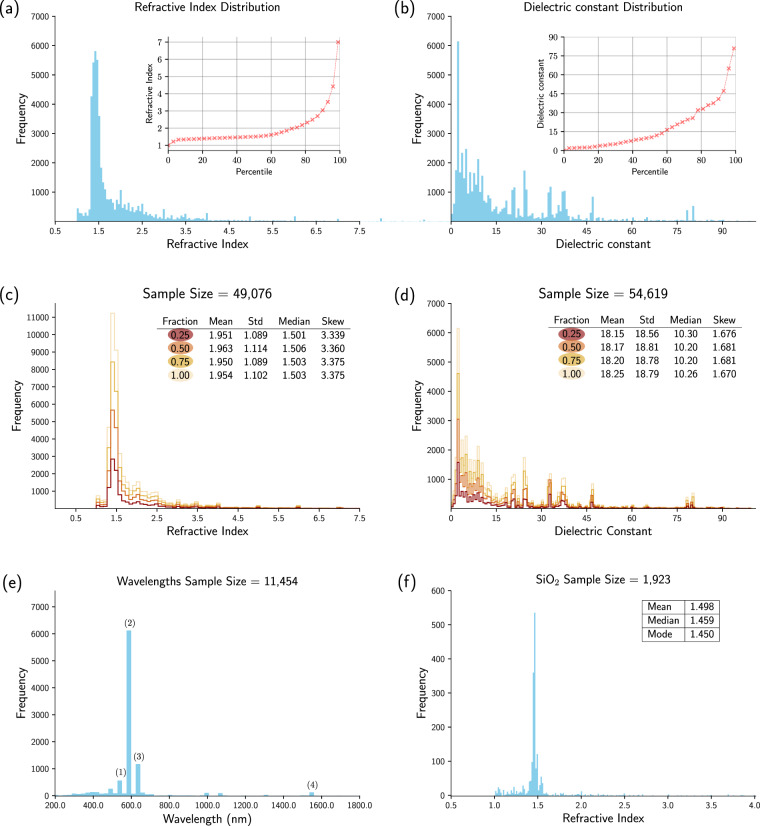
Data validation. (**a**) Histogram of experimental refractive index values for all valid records in the database (inset: experimental refractive index percentiles). (**b**) Histogram of experimental dielectric constant values for all valid records in the database (inset: experimental dielectric constant percentiles). (**c**) Histogram and statistics for different fractions of the database, drawn randomly from all valid records of the experimental refractive-index values. (**d**) Histogram and statistics for different fractions of the database, drawn randomly from all valid records of the experimental dielectric-constant values. (**e**) Histogram of all wavelengths associated with a refractive index in the database. (**f**) Histogram and statistics of all refractive index values of SiO_2_ in the database.

The generalisability of the database can be seen by visualising relevant histograms for each set of properties; see Fig. 3. The distribution of 49,076 valid refractive index records with a value ≤ 7.5 is shown in Fig. 3a by a blue histogram that is split into 200 bins. The vast majority of data sit between 1.3 and 1.8, and ~ 80% of the data lie between 1 and 2, which can be seen from the inset percentile plot. The peaks at 1.5, 2.7 and 3.4 reveal the wide use of SiO_2_, TiO_2_ and Si in modern optical industry applications. Similarly, the distribution of 54,619 valid dielectric constant records with a value ≤ 100 is shown in Fig. 3b. The dielectric-constant distribution is less concentrated than that of the refractive index, owing to a wider variety of its measurement condition; for example, the measurement frequency usually varies from Hz to THz.

The database appears to contain a representative set of each property. This was deduced by comparing distribution statistics for each of the two properties against those from three randomly sampled data subsets that contain 25%, 50% and 75% of the total data set. Histograms of the resulting distributions are shown in Fig. 3c for the refractive index and Fig. 3d for the dielectric constant. Mean, standard deviation, median and skewness were calculated to serve as quantitative evaluation metrics for these comparisons and are shown in the inset tables. Visual inspection of these results clearly indicates that the essential features of each histogram are preserved.

The nested parsing logic of ChemDataExtractor allows the extraction of additional information from a document where it is mentioned together with a valid record in the same sentence. For the refractive index data set, 11,454 out of the 49,076 refractive index records were mentioned together with information about its measurement wavelength. Figure 3e displays the distribution of these 11,454 measurement wavelength data. Most of the wavelength data lie within the visible spectrum which suggests that the visible spectrum is currently the most popular wavelength range for research into refractive index applications. Peak (1) at 550 nm occasionally occurs. This is possibly because 550 nm is close to the maximum of the response curve in human photopic vision. The sharp Peak (2) at 589 nm corresponds to the ‘sodium D-line’; this likely reveals a wide use of Abbe refractometers in the modern measurement of refractive indices, since 589 nm is the standard measurement wavelength for most Abbe refractometers. Peak (3) at 633 nm lie in the red part of the visible spectrum and is the wavelength of a Helium–Neon laser. This suggests that many refractive index measurements were carried out using a Helium–Neon laser, which stands to reason given its relatively low cost and ease of operation. Peak (4) at 1,550 nm corresponds to a minimum-loss window for telecommunication applications; as such, it corresponds to the working wavelength of single-mode optical fibres.

The database exhibits a certain level of redundancy for two reasons. On the one hand, two parsers were used to extract text and two parsers were employed to extract data from tables; so there may be an overlap in data extraction. On the other hand, the same {material, property} relationship may be mentioned more than once in an article. However, if the relationship within one sentence or in one table cell was extracted twice, independently by two different methods, it is reasonable to infer that the probability of this relationship being positive is higher; the probability of this relationship being negative is $(1-{\rm{P}}1)\times (1-{\rm{P}}2) < (1-{\rm{P}}1)\;{\rm{or}}\;(1-{\rm{P}}2)$, where P1 and P2 are the true precision of method 1 and 2. Thus, the redundancy of the database is preserved. An example of how the redundancy may help the downstream analysis of this database is explained in Fig. 3f. Figure 3f shows the histogram of all 1,928 refractive index records of SiO_2_ in our database. The distribution peaks sharply at 1.46 which refers to the refractive index of fused silica measured at 589 nm, at room temperature^21^. More than 80% of data lie within 1.40–1.55 which corresponds to the refractive index of fused silica measured between 210 nm to 3,710 nm at room temperature^21^. The mean, median, and mode of these data all lie within 1.45–1.50 and vary from 1.46 by at most 2.6%. Records beyond 1.40–1.55 are likely to be false positives, thereby reducing the precision of the database as a whole. However, this example shows that users can eliminate many of the incorrect records by employing data-aggregation methods on a given material in the downstream analysis of a database.

To this end, our database auto-generation methods have demonstrated their potential to afford a database that is likewise to be used in data-driven materials discovery for optical applications. For instance, a user may wish to use this database as a source to develop a data-driven “design-to-device” operational pipeline^22^. Successful studies that have generated and used auto-generated databases via similar efforts to this work have been reported in recent papers^13,23–26^, whereby a small short-list of leading candidates can be progressively filtered down from the database for a target material application^22,23,27^. In such scenarios, false positive database entries would likely be filtered out naturally during downstream analysis, while a data source that carries a large amount of data is essential for such a data-driven task. Also, a user may need a simple “look-up” database in which quality control is imperative but the property sought is common. In such a scenario, precision is valued over the quantity of data. The “Warning” data field (see Data Records) in our database will help the user to better process the database according to their specific purpose. Users who would like to obtain access to rarely mentioned compounds can easily find them through the warning flag “S”. Users should pay particular attention to the data with a warning flag “O” as they are more likely to be measured under abnormal external conditions or are incorrect data records.

In the general case, the value of the optical constants should be described in full, in the form of complex number, and they also depend on a range of external parameters such as experimental conditions and the 3-D structures of the material. At present, we are able to identify some measurement wavelength information of the refractive index and limited frequency information of the dielectric constant, where papers describe them within the same sentences. However, the number of such results (ca. 11,517 data records) is small compared to the total number of data (ca. 10%). Due to these simplifications and limitations, the database is more likely to be used for data-driven application purposes rather than a strict error-free database for experimentalists. With further development of our software tools, we plan to continuously maintain our database and work on these limitations in a future stage of our research.

## Usage Notes

The database has been made available in both relational and non-relational formats including CSV, JSON and SQL. The flexibility of its data structure enables the database to be easily read, queried, and updated by database languages such as SQL or Mongo, as well as programming languages such as Python and MATLAB. The database can be found at^19^. We have also provided a user-friendly website (www.opticalmaterials.org) for users to view, query and download the database. Users can directly type in the compound name, property specifier, DOI, or a certain range of values in a search box to look for target materials. Users are also able to view the distribution and statistics of the search results. The Python codes that were used to generate the database and the modified version of ChemDataExtractor can be found at https://github.com/JIUYANGZH/opticalmaterials_database. This pipeline can also be used and adjusted for the data extraction of other interested material properties.

## Supplementary information

Supplementary Information S1

Supplementary Information S2

Supplementary Information S3

Supplementary Information S4

Supplementary Information S5

## Data Availability

The source code used to generate the database is available at https://github.com/JIUYANGZH/opticalmaterials_database. The code of ChemDataExtractor that was modified for database auto-generation in the optical-property domain is available at https://github.com/JIUYANGZH/opticalmaterials_database/tree/master/chemdataextractor. The source code of the website can be found at https://github.com/JIUYANGZH/aws_test.
