# Supplementary material for: A database of refractive indices and dielectric constants auto-generated using ChemDataExtractor
Source: Sci Data. 2022 May 3;9:192. doi: 10.1038/s41597-022-01295-5 (PMC9065060; doi:10.1038/s41597-022-01295-5)
Supplement: Supplementary file 5 — Supplementary Information S5 [file 41597_2022_1295_MOESM5_ESM.pdf]

Supplementary Information for:

A database of refractive indices and dielectric constants auto-generated using ChemDataExtractor

Jiuyang Zhao[1], Jacqueline M. Cole[1,2,3*]

1. Cavendish Laboratory, University of Cambridge, J. J. Thomson Avenue, Cambridge, CB3 0HE, U.K.

2. ISIS Neutron and Muon Source, Rutherford Appleton Laboratory, Harwell Science and Innovation Campus, Didcot, Oxfordshire, OX11 0QX, UK.

3. Department of Chemical Engineering and Biotechnology, University of Cambridge, West Cambridge Site, Philippa Fawcett Drive, Cambridge, CB3 0AS, U.K.

*Corresponding author(s): Jacqueline M. Cole (jmc61@cam.ac.uk)

Table of Contents

S1 – Sampled dataset for the refractive index precision validation .... (*separate file*)

S2 – Sampled dataset for the dielectric constant precision validation................................. (*separate file*)

S3 – Sampled dataset for the refractive index recall validation.. (*separate file*)

S4 – Sampled dataset for the dielectric constant recall validation....................................... (*separate file*)

S5 – Legend for the error type abbreviated in the precision validation datasets 2

S5 - Precision Validation Legend – Correctness

CO1 – Coordination error type I, wrong match. For example, "A and B have refractive index of C and D." Wrong match means A-D and B-C were extracted instead of A-C and B-D.

CO2 – Coordination error type II. One of the entities was not extracted. For example, "A and B have refractive index of C and D.", only A and D were extracted, and D was assigned to A to be A-D.

IN – Incorrect name. It usually comes from failures of chemical named entity recognition system. It could be a non-chemical been recognized as a chemical, or a chemical name was only partially extracted.

TCH – TableDataExtractor error. It usually refers to cases that the value being assigned to the compound in another (wrong) row. Or values in a wrong column were extracted.

WS – Wrong specifier. It normally refers to cases that a word that is not the property specifier been identified as a specifier, or the existence of ambiguous words. For example, "n" is a property specifier of refractive index, but it can refer to kinetic parameter, carrier density etc.

WN – Wrong number. It usually refers to cases that a scientific notation been not properly extracted or numbers with a logarithm symbol, e.g. ln2.

PP – Property parser error. It refers to the mistakes in the property parser rules. It could be both human error and systematic errors due to the complexity of parser rules.

RD – Reader error. For some old papers, e.g. papers published in 1970s. The electronic version may not be formatted in a standard format. Thus, the document reader can make mistakes to read these old papers. Also, some papers are only available to be downloaded in the PDF format and the PDF reader of ChemDataExtractor is still under development.

SB Table – Special binary table. For some papers that describing physical property of binary systems, the table format is less decoding-friendly, which it is very often that only part of the binary system was extracted.

UN – Unrecognized name. Failure of chemical named entity recognition system. A chemical name was not extracted while it should be.
